# Toxicology Evaluation of Realgar-Containing *Niu-Huang-Jie-Du Pian * as Compared to Arsenicals in Cell Cultures and in Mice

**DOI:** 10.5402/2011/250387

**Published:** 2011-10-13

**Authors:** Jia-Wei Miao, Shi-Xia Liang, Qin Wu, Jie Liu, An-Sheng Sun

**Affiliations:** ^1^Key Lab of Basic Pharmacology, Zunyi Medical College, Zunyi 563000, China; ^2^Chongqing Three Gorges Medical College, Chongqing 404120, China; ^3^University of Kansas Medical Center, Kansas KS 66160, USA

## Abstract

*Niu-Huang-Jie-Du Pian (NHJD)* is a widely used traditional Chinese medicine containing realgar (As_4_S_4_). Realgar has been included in many traditional medicines, but is often taken as arsenite for risk assessment. To evaluate true risk of realgar and realgar-containing *NHJD*, their toxicity was compared with common arsenicals. In cultured cells, the LC_50_ for *NHJD* (1200 *μ*M) and realgar (2000 *μ*M) was much higher than arsenite(35 *μ*M), arsenic trioxide (280 *μ*M), and arsenate (400 *μ*M). Acute toxicity in mice showed more severe liver and kidney injury after arsenite or arsenate, but was mild after realgar and *NHJD*, corresponding to cellular and tissue arsenic accumulation. The expressions of arsenic-sensitive stress gene metallothionein-1 were increased 3–7-folds after arsenite or arsenate, but were unaltered after *NHJD* and realgar. Thus, realgar and *NHJD* are much less toxic than arsenite and arsenate. The use of total arsenic to evaluate the safety of realgar and realgar-containing *NHJD* is inappropriate.

## 1. Introduction

Realgar (90% as As_4_S_4_) has been used in traditional Chinese medicines [1, 2] and in Indian Ayurvedic medicines [3] for thousands of years and claimed to have therapeutic effects in these remedies. However, arsenic (As) is a highly toxic substance and its risk in traditional remedies is of concern [4–6]. Hundreds of traditional medicines are forbidden in the USA or European market because of the contents of As are higher than the allowable limits for food and drugs [2], and over 20% of online-sold Ayurvedic medicines were demanded for rigorous regulation for heavy metal contents [6]. The Chinese Pharmacopeia Committee has reduced the allowable As contents in traditional medicine recipes by as much as 65%, but As contents are still thousands-fold over the health food standards. More studies are therefore recommended to evaluate the true risk of metal-containing traditional medicines [7].

Niu-Huang-Jie-Du Pian (*NHJD,* **牛黄解毒片**) is a popular realgar-containing traditional Chinese medicine (http://www.wickpedia.org/), which can be easily obtained in Chinese grocery stores worldwide. *NHJD* is composed of realgar (6.4%), Niu-Huang (*Calculus bovis*), Huang-Qin (*Radix scutellariae*, rhubarb), Ju-Geng (*Platycodon grandiflorum*), Gan-Cao (*Radix glycyrrhizae uralensis*, licorice root), gypsum (calcium sulfate), and Bing-Pian (*Borneol*), and it is used for antipyretic, cold, gingivitis and other inflammatory diseases [1]. There is a general perception that the use of toxic metals in medicines is an unacceptable risk, but an opposing opinion holds that realgar-containing traditional medicines are not necessarily toxic at clinical doses [2, 7]. 

We have recently shown that chemical form of realgar (As_4_S_4_) is a major determinant for its disposition and toxicity. For example, realgar-containing An-Gong-Niu-Huang Wan (*安宫牛黄丸*) was much less toxic in cultured cells [8], in acute animal studies [9], and in subchronic toxicology studies [10, 11]. The present study was undertaken to evaluate the true risk of a realgar-containing *NHJD*, a most popular patent Chinese medicine, in cultured cells and in intact animals, as compared to common arsenicals. The results fortified our prior conclusions that chemical forms of metals are very important in determining the disposition and toxicity of metal-containing traditional medicines.

## 2. Materials and Methods

### 2.1. Chemicals

Realgar (>90% of As_4_S_4_), and realgar (6.4%)-containing *NHJD *were obtained from Beijing Tong-Ren-Tang Technologies Co., Ltd., Beijing, China; The common arsenicals As_2_S_2_, As_2_O_3_, NaAsO_2_, and Na_2_HAsO_4_·7H_2_O were purchased from Sigma Chemical Company (St. Louis, Mo, USA), all chemicals were of reagent grade.

### 2.2. Cell Cultures and Treatments

Human pharyngeal carcinoma FaDu cells were obtained from Shanghai Institute of Biochemistry and Cell Biology (Shanghai, China) and cultured in DMEM media supplemented with 10% fetal bovine serum (FBS) with penicillin and streptomycin. Cells were cultured at 37°C in a 5% CO_2_-humidified atmosphere. All chemicals were dissolved in dimethyl sulfoxide (DMSO) in 10–30 mM and serially diluted in culture media before addition to the cultures at 5-6 different concentrations as indicated. For cytotoxic assay, chemicals were added to 96-wells when initiating the culture, and 48 h later, the cytotoxicity was measured by the MTS assay as described previously [8]. For As uptake and accumulation studies, cells were exposed to the same concentration (100 *μ*M) for 30–120 min, and cellular As was determined after thoroughly washing.

### 2.3. Animals and Treatments

Adult male and female Kunming mice (22 ± 2 g) were purchased from the Animal Center of the Third Military Medical University (Chongqing, China). Mice were kept in a regulated environment (22 ± 1°C, 50 ± 2% humidity) with a 12 h : 12 h light:dark cycle. All animal procedures follow the WHO Guidance of Humane Care and Use of Laboratory Animals. 

Mice were orally administered with *NHJD* (600 mg/kg), realgar (600 mg/kg), and the equal amount of As doses as sodium arsenite (36 mg/kg) and sodium arsenate (88 mg/kg). The NHJD 600 mg/kg is approximately 2 times of clinical dose (2000 mg/day/60 kg person, taken mouse-human extrapolation factor of 10). Animals were monitored closely for clinical symptoms after gavage, and tissues were harvested for analysis 8 hrs later. The doses of arsenicals selection were based on our recent publications [9, 12].

### 2.4. Blood Biochemistry

Serum was separated from whole blood by standing for 1 hr, and blood biochemistry was determined with an autoanalyzer (GLAMOUR1600). The serum activities of alanine aminotransferase (ALT) and concentrations of blood urea nitrogen (BUN) were quantified to evaluate the hepatotoxicity and nephrotoxicity of animals treated with *NHJD* and various arsenicals.

### 2.5. Arsenic Determination

Total arsenic contents in cells and tissues were analyzed by atomic fluorescence spectrometry (AFS) as described previously [12]. Briefly, tissues were completely digested in nitric acid at 170°C for 2.5 hrs and brought to 25 mL with distilled water, and 5 mL of the sample was mixed with 1 mL 5% thiourea-ascorbic acid solution. Following 30 min incubation, aliquots were used for quantification of As contents with atomic fluorescence spectrometry (Kechuang Haiguan Instrument Co. Ltd, Beijing, China). These assays were performed at the Guizhou Chemical Analysis Center of Chinese Academia of Sciences [12].

### 2.6. RNA Isolation and Real-Time RT-PCR Analysis

RNA isolation and real-time RT-PCR analysis approximate 50–100 mg tissue was homogenized in 1 mL TRIzol agent (Invitrogen, Carlsbad, Calif, USA), and total RNA was extracted according to manufacturer's instructions, followed by purification with RNeasy columns (Qiagen, Valencia, Calif, USA). The quality of RNA was determined by the 260/280 ratios, and by gel electrophoresis to visualize the integrity of 18S and 28S bands. Total RNA was reverse transcribed with MMLV reverse transcriptase and oligo-dT primers. The PCR primers were designed with Primer Express software (Applied Biosystems, Foster City, Calif, USA) as MT-1 (BC027262), forward: AATGTGCCCAGGGCTGTGT; reverse: GCTGGGTTGGTCCGATACTATT. The Power SYBR Green Mater Mix (Applied Biosystems, Foster City, CA, USA) was used for real-time RT-PCR analysis. The cycle threshold (Ct) values of the interested genes were first normalized with *β*-actin of the same sample and expressed as percentage of controls.

### 2.7. Statistical Analysis

For cytotoxicity analysis, means and standard error were calculated from 5 separate cultures, and the LC_50_ values were estimated via graphics. For animal studies, means and standard error of 6 mice were calculated. Data were analyzed using a one-way analysis of variance (ANOVA), followed by Duncan's multiple range test. The significant level was set at *P* < 0.05 in all cases.

## 3. Results

### 3.1. Cytotoxicity of NHJD and Arsenicals in Human Pharyngeal FaDu Cells

**Figure 1:**
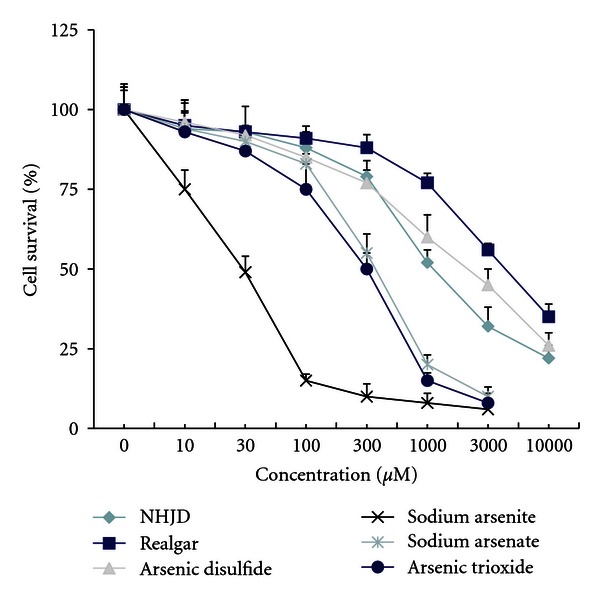
Human nasopharyngeal carcinoma FaDu cells were exposed to chemicals for 48 h and toxicity was determined by the MTS assay. The rank orders of LC_50_ are sodium arsenite (35 *μ*M) > arsenic trioxide (280 *μ*M), arsenate (400 *μ*M) >*NHJD* (1200 *μ*M) > realgar (As_2_S_2_, 2000 *μ*M; As_4_S_4_, 3000 *μ*M). Data are mean ± SE of 5 separate experiments.

Differential cytotoxicity between realgar-containing An-Gong-Niu-Huang Wan and arsenicals was evident in human nasopharyngeal carcinoma FaDu cells [8]. Thus, the cytotoxicity potential of *NHJD* and arsenicals was examined in this cell line. Figure 1 illustrated that the LC_50_ at 48 h for realgar-containing *NHJD* was 1200 *μ*M, for realgar was 2000 *μ*M. The most toxic arsenicals was sodium arsenite (35 *μ*M), sodium arsenate (400 *μ*M), arsenic disulfide (As_2_S_2_, 2000 *μ*M), and arsenic trioxide (As_2_O_3_, 280 *μ*M).

### 3.2. Accumulation of Arsenic in Human Pharyngeal FaDu Cells

**Figure 2:**
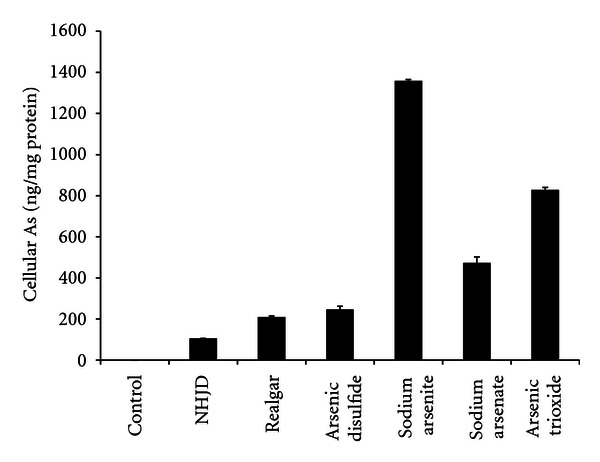
Cellular As accumulation. FaDu cells were exposed to 100 *μ*M of arsenicals for 30 min. After washing 3 times in PBS, cells were harvested; cellular protein determined and As contents were determined by atomic fluorescence spectrometry (AFS). Data are mean ± SE of 3 separate experiments.

Confluent FaDu cells were exposed to *NHJD* and arsenicals, all at the 100 *μ*M concentrations for 30 min, and cultures were thoroughly washed and cellular uptake of As were determined as described in Methods. The results (Figure 2) show dramatic difference in cellular arsenic accumulation, *NHJD,* and realgar treatments resulted in 100–200 ng As/mg cellular protein, while sodium arsenite and sodium arsenate resulted in 1360 and 470 ng As/mg protein, respectively. Similar results were also obtained after 60 min and 120 min exposure (data not shown).

### 3.3. Blood Biochemistry of NHJDP and Arsenical Treatments in Mice

Mice were orally administered with *NHJD* (600 mg/kg), realgar (600 mg/kg, equivalent to reaglar in *NHJD*), and the equal amount of As doses of sodium arsenite (36 mg/kg) and sodium arsenate (88 mg/kg). Animals were killed 8 hr later and blood biochemistry was performed. The results (Table 1) show that the elevations of serum ALT and BUN after arsenite and arsenate, but these parameters were unaltered after *NHJD,* and realgar. Histopathology was consistent with blood biochemistry, showing more severe liver and kidney damage after arsenite and arsenate, but mild or absent after *NHJD* and realgar [13].

### 3.4. Accumulation of Arsenic in Liver and Kidneys after NHJD and Arsenical Treatments in Mice

**Figure 3:**
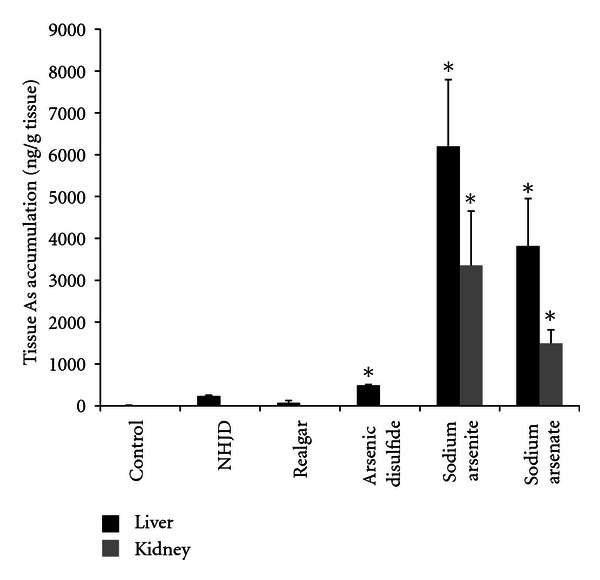
As accumulation in liver and kidney. Mice were orally given *NHJD* (600 mg/kg), realgar (600 mg/kg), arsenic disulfide (600 mg/kg), sodium arsenite (36 mg/kg), or sodium arsenate (88 mg/kg). Tissues were collected 8 h later for analysis by atomic fluorescence spectrometry (AFS). Data are mean ± SE of 6 mice and expressed as ng As/g wet tissue. *Significantly different from controls *P < 0.05.

Mice were orally administered with *NHJD* (600 mg/kg), realgar (600 mg/kg, equivalent to realgar in *NHJD*), and the equal amount of As doses of sodium arsenite (36 mg/kg) and sodium arsenate (88 mg/kg). Animals were killed 8 hr later and tissue As accumulation was determined. The results (Figure 3) show that the dramatic difference in tissue arsenic accumulation, *NHJD* and reaglar treatments resulted in approximately 200 ng As/g liver, while arsenite (6200 ng/g liver) and arsenate (3320 ng/g liver) produced significant As accumulation. Renal As contents were under detection limits for *NHJD* and realgar, but reached 3350 ng/g kidney after arsenite and 1500 ng/g kidney after arsenate.

### 3.5. Expression of Metallothionein-1 in Liver and Kidneys

**Figure 4:**
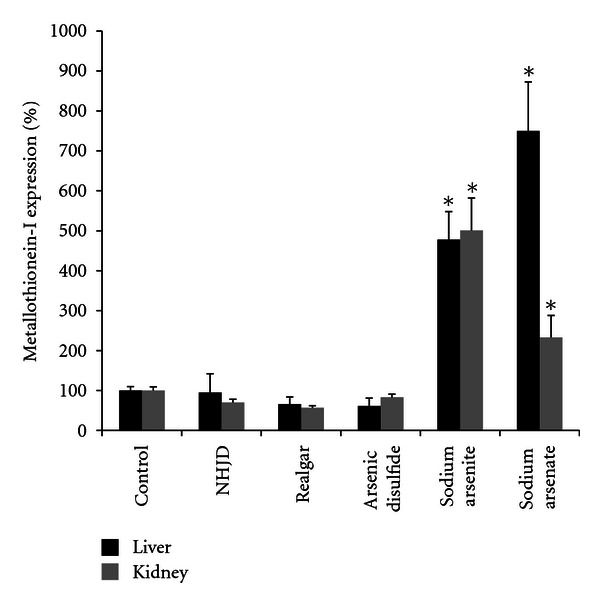
Relative transcript levels of metallothionein-1 (MT-1) in liver and kidney. Mice were orally given *NHJD* (600 mg/kg), realgar (600 mg/kg), arsenic disulfide (600 mg/kg), sodium arsenite (36 mg/kg), sodium arsenate (88 mg/kg), or distilled water (Control). Tissues were collected 8 h later for total RNA isolation, followed by real-time RT-PCR analysis. Data are mean±SE of 6 mice. *Significantly different from controls *P* < 0.05.

Figure 4 shows the expression of metallothionein-1 (MT-1) in liver and kidney. MT is a small, cysteine-rich, metal-binding protein playing an important role in metal detoxication [14]. MT overexpression is a sensitive biomarker for arsenic-induced stress [15]. As shown in Figure 4, hepatic MT-1 was increased 5–7-folds after arsenite and arsenate, but was not altered after *NHJD *and realgar. As shown in Figure 4, bottom, renal MT-1 transcript levels were also increased after arsenite (5-fold) and arsenate (2-fold), respectively. In comparison, *NHJD* and realgar did not produce significant elevation in renal MT-1 transcripts.

## 4. Discussion

The present study clearly demonstrated that realgar and realgar-containing *NHJD *were much less toxic than sodium arsenite and arsenate in cultured cells and in mice, as evidenced by LC_50_ values, the elevated serum ALT and BUN concentrations in mice. Toxicokinetically, much less As was accumulated in the cells or in tissues after realgar and *NHJD*. Furthermore, the expression of stress-related genes, namely, MT-1, was increased 2–7-folds after sodium arsenite and arsenate in the liver and kidneys, but was basically unaltered after realgar and *NHJD,* consistent with tissue damage. The present study fortified our recent observations in cultured cells [8] and in intact animals [9–13], indicating that realgar and realgar-containing *NHJD* are different from sodium arsenite and arsenate and clearly demonstrate that the chemical forms of arsenicals underlies their disposition and toxicity potentials. 

Arsenic has been used as a remedy and a poison since ancient times. In addition to the use of arsenic compounds in cancer chemotherapy [16, 17], realgar has been included in 22 oral patent traditional remedies according to Pharmacopeia of China [1], and was claimed to have many beneficial effects for various diseases [1–3, 16, 17]. However, the major concern is their toxicity potential, which limits many realgar-containing remedies. *NHJD* is a most popular realgar-containing traditional Chinese medicine (http://www.wickpedia.org/) available in Chinese grocery stores in the USA and in the Europe and is used for many inflammatory diseases [1]. To critically evaluate its toxicity potential is important for safely use of realgar and realgar-containing traditional medicines in the treatment of various inflammatory diseases.

Arsenic exists in the trivalent (As^3+^, arsenite) and pentavalent (As^5+^, arsenate) forms and is widely distributed in nature. In general, sodium arsenate (LD_50_ 112–175 mg/kg) is 4-5 times less acutely toxic than sodium arsenite (LD_50_ 15–44 mg/kg), and the pentavalent organic arsenicals MMA, DMA, and TMA are 40–100 times less acutely toxic than arsenite [18]. Arsenicals in seafood mainly exist as organic forms, such as arsenobetaine, arsenosugar, and arsenocholine, with acute oral LD_50_ values 100–500-fold above arsenite or arsenate [2]. In traditional medicines, the oral LD_50_ for arsenic trioxide (i.e., arsenolite) in mice is 32–39 mg/kg, but the LD_50_ for realgar is 3.2 g/kg [2], a 100-fold difference in acute toxicity. Thus, arsenical toxicity is highly dependent on the chemical form, and realgar (As_4_S_4_) is much less acutely toxic than arsenic trioxide (As_2_O_3_) and is also much less acutely toxic than sodium arsenite (NaAsO_2_) and arsenate (Na_2_HAsO_4_) in the present study. 

It is generally assumed that the severity of poisoning is related to the total amount of poison ingested, and assessment of health risk associated with arsenic exposure from human ingestion of traditional medicines has typically taken this tactic [4–7]. However, the present study clearly showed that realgar was poorly accumulated into the cells and tissues and was unable to reach a critical concentration to cause tissue damage as compared to the same amount of arsenic given as arsenite and arsenate, with tremendous differences in their tissue contents. The disposition of these arsenicals in the body depends on various key factors including solubility, absorption, distribution, and excretion [2]. For example, the average total arsenic in *NHJD* is about 7 ± 1% (i.e., 70,000 ppm), corresponding to 28 mg arsenic per pill, of which only 1 mg arsenic finds its way into the blood stream, that is, only 4% of intake realgar is bioavailable [19]. Compared to realgar, 80% of orally given sodium arsenite and arsenate can be absorbed from the gastrointestinal tract, with much higher plasma As levels and tissue distribution [2, 18]. The bioavailability is a critical determinant of efficacy and toxicity of arsenical compounds. Thus, it is the amount of toxicants to the target organ, rather than the amount ingested or inhaled that makes a poison [20].

Various stresses have been proposed as an important mechanism involved in arsenic toxicity and carcinogenesis. MT is a small, cysteine-rich, metal-binding protein playing an important role in metal toxicity [14]. Induction of MT by arsenicals is dependent on arsenic forms, with sodium arsenite is the most potent and efficient inducer followed by arsenate and organic arsenicals, such as MMA and DMA [21], and MT-null mice were susceptible to chronic arsenic-induced hepatotoxicity and nephrotoxicity [22]. MT induction is considered an adaptive mechanism to toxic metal-induced oxidative stress [14, 15] and can be used as an indicator for arsenic-induced stress. In the present study, hepatic and renal MT was induced by sodium arsenite and arsenate, but was basically unaltered by realgar and *NHJD*, fortifying the observations from blood biochemistry that realgar and *NHJD *are much less acutely toxic than sodium arsenite and arsenate.

Traditional medicines are based on empirical experience and have their own theory and are generally safe at clinical doses. The regulation of metal-containing traditional medicines has been a topic of debate [2–7, 13, 23–25]. We have been invited to write two reviews on this topic for arsenic (J Pharmcol Exp Ther [2]) and for Hg (Exp Biol Med [26] and have challenged this bias by studying the relative safety of Liu-Shen-Wan (*六神丸*,  As) [12], An-Gong-Niu-Huang-Wan (*安宫牛黄丸*,  As+Hg) [9–11], and Zhu-Sha-An-Shen-Wan (*朱砂安神丸*,  Hg) [27]. In the present study, *NHJD* is unique in that it is the most popular traditional medicine available not only in China [1], but also in Chinese grocery stories worldwide. To study its relative safety is of clinical significance. 

Realgar is less toxic than arsenic oxide yet effective in cancer chemotherapy based on the recent literature [23]. However, this does not imply that realgar and *NHJD* are nontoxic. High dose of realgar for the long-term use did produce toxicity to the liver and kidneys [24], similarly, long-term use *NHJD *was reported to produce hepatotoxicity and nephrotoxicity [25]. In the evaluation of realgar and realgar-containing *NHJD *in the treatment of various diseases, the balance of the benefit and risk is important. “Dose makes a poison.” Although realgar and *NHJD* are less toxic than sodium arsenite and arsenate, high-dose and long-term administration should be avoided to reduce undesired adverse effects and toxicity [2, 18]. 

In summary, the present study clearly demonstrated that realgar and realgar-containing *NHJD* are much less toxic to the cultured cells and to the intact animals, as compared to sodium arsenite and arsenate. The chemical forms of arsenicals determine their tissue accumulation and toxicity potentials, and thus the use of the total content of As to evaluate realgar-containing traditional medicines is inappropriate.

## Figures and Tables

**Table 1:** Serum ALT and BUN concentrations in mice treated with NHJD and arsenicals.

Groups	Dose	ALT (U/L)	BUN (mmol/L)
Control	0	45.3 ± 5.1	11.3 ± 0.1
NHJD	600 mg/kg	64.3 ± 18.2	13.6 ± 2.2
Realgar	600 mg/kg	52.8 ± 12.1	11.2 ± 2.3
Arsenic disulfide	600 mg/kg	58.2 ± 9.0	11.9 ± 1.0
Sodium arsenite	36 mg/kg	103 ± 31.4*	15.6 ± 3.0*
Sodium arsenate	88 mg/kg	92.5 ± 18.3*	14.2 ± 1.7*

Data are mean ± SE, *n* = 6. *P < 0.05.

## Abbreviations

*NHJD:*: Niu-Huang-Jie-Du Pian (*牛黄解毒片*), a realgar-containing traditional medicine
MTS: [(3-(4,5-dimethylthiazol-2-yl)-5-(3-carboxymethoxyphenyl)-2-(4-sulfophenyl)-2*H*-tetrazolium)
MT-1: Metallothionein-1
As: Arsenic
Hg: Mercury.
